# Burnout in Pediatric Nephrology Fellows and Faculty: Lessons From the Sustainable Pediatric Nephrology Workforce Project (SUPERPOWER)

**DOI:** 10.3389/fped.2022.849370

**Published:** 2022-05-04

**Authors:** Susan M. Halbach, Kartik Pillutla, Patricia Seo-Mayer, Alan Schwartz, Darcy Weidemann, John D. Mahan

**Affiliations:** ^1^Division of Nephrology, Seattle Children's Hospital, Seattle, WA, United States; ^2^Department of Pediatrics, University of Washington, Seattle, WA, United States; ^3^Department of Pediatrics, University of Texas Dell Medical School, Austin, TX, United States; ^4^Division of Nephrology, Inova Children's Hospital, Falls Church, VA, United States; ^5^Department of Medical Education and Department of Pediatrics, University of Illinois at Chicago, Chicago, IL, United States; ^6^Association of Pediatric Program Directors, McLean, VA, United States; ^7^Division of Nephrology, Children's Mercy Kansas City, Kansas City, MO, United States; ^8^Department of Pediatrics, University of Missouri—Kansas City School of Medicine, Kansas City, MO, United States; ^9^Division of Nephrology and Hypertension, Nationwide Children's Hospital, The Ohio State University, Columbus, OH, United States

**Keywords:** workforce, burnout, pediatric nephrology, fellow, faculty

## Abstract

Physician well-being is an important contributor to both job satisfaction and patient outcomes. Rates of burnout among physicians vary by specialty, ranging from 35 to 70%. Among pediatric residents, longitudinal data demonstrates consistent rates of burnout around 50-60%, although little is known about burnout among pediatric subspecialty fellows. Specifically, the degree of burnout among pediatric nephrologists remains unknown, as does the impact faculty burnout may have on trainee burnout. We sought to evaluate prevalence and predictors of burnout among US pediatric nephrology fellows and faculty, and assess for interactions between groups. In this multi-center pilot survey of all United States pediatric nephrology training programs from February to April 2020, burnout was assessed through abbreviated Maslach Burnout Inventory and predictors were explored through survey items devoted to demographic, personal characteristics, and job and career satisfaction questions. A total of 30/34 available fellows and 86/102 faculty from 11 institutions completed the survey (overall response rate 85%). The prevalence of burnout was 13% among fellows and 16% among faculty. Demographic (age, gender, year of training, faculty rank, marital status) and program factors (fellowship size, faculty size, current block/rotation, vacation or weekend off timing) were not significantly associated with burnout. Faculty and fellows with burnout reported significantly lower quality of life (5.3 vs. 7.9, *p* < 0.05), higher perceived stress (2.4 vs. 1.4, *p* < 0.05) and lower satisfaction with career choice (66 vs. 22%) and work life balance (28 vs. 0%), compared to those without burnout (*p* < 0.05 for all). Other important factors positively associated with burnout included lower institutional support for wellness programs and lower satisfaction with both colleague and faculty support. Larger studies are needed to explore if burnout is truly less prevalent among pediatric nephrology fellows and faculty compared to pediatric residents and graduate physicians. A larger sample size is also necessary to determine whether any interactions exist between the faculty and trainee roles in the developments of burnout. Future studies should also explore how to promote well-being through addressing key factors such as overall learning/working environment, stress reduction, and building personal resilience.

## Introduction

The prevalence of burnout is substantially higher among US physicians than in the general population (40 vs. 28%), although rates vary by specialty (35-70%) (1, 2). Among US pediatric nephrologists, the prevalence of burnout and job satisfaction is unknown, as previous studies group pediatric medical specialties together. In terms of trainee burnout, longitudinal data suggests a consistent prevalence of 50-60%, but little is known about pediatric subspecialty fellows (3). Physician burnout is not only harmful for the individual physician, but has been associated with higher rates of stress, depression and suicidal ideation as well as higher rates of employee turnover and poor patient outcomes (2, 4).

While the impact of faculty stress and levels of burnout on fellows has not been well explored, Prior studies evaluating the impact of organizational leadership and satisfaction with divisional/departmental supervisors on physician burnout have found increased stress and burnout among physicians who perceive their leaders as less effective (5, 6). However, the impact of faculty stress and burnout on fellow trainees has not been explored.

The primary objective of our study was to determine the prevalence of burnout among pediatric nephrology fellows and faculty and to explore evidence that burnout levels are higher in fellows exposed to faculty with higher levels of burnout. Additionally, we sought to assess significant predictors of burnout at the individual, program and institutional levels in these two groups by assessing demographics, program and personal characteristics, career satisfaction, and attitudes. Finally, we compared responses from trainees and faculty within each institution.

## Methods

### Participants

We conducted a multicenter survey of pediatric nephrology fellows and faculty through the American Association of Pediatric Program Directors Longitudinal Educational Assessment Research Network (APPD LEARN). Out of 46 ACGME-accredited pediatric nephrology training programs, 42 APPD-member pediatric nephrology programs were eligible to participate based on membership in APPD. Study recruitment occurred in two phases. First, program directors of all eligible programs were invited to participate as study site liaisons through email and verbal communication at subspecialty society meetings. Each participating program obtained local Institutional Review Board approval. Second, all fellows and faculty at participating programs were invited to complete the anonymous survey. There were no incentives provided to participate and study site liaisons did not have access to either the survey responses nor information on who completed the survey.There were no exclusion criteria.

### Survey Content and Design

The survey consisted of 90 items in the following domains: demographic, program-level and division-level information, abbreviated Maslach Burnout Inventory (MBI), career satisfaction questions, and assessment of sleep, stress, self-compassion and resilience through previously validated survey instruments used in previous studies from the Pediatric Resident Burnout—Resilience Study Consortium (7). The abbreviated MBI consisted of two questions assessing the elements of emotional exhaustion and depersonalization: “I feel burned out from my work” and “I've become more callous toward people since I took this job.” Response options were on a seven-point rating scale ranging from “never” to “every day.” In this method, burnout was defined as either or both of the two items being endorsed with frequency “once per week” or more. Previous work with healthcare professionals has demonstrated concordance of the 2-question burnout assessment to the full MBI results (8).

### Data Collection and Analysis

The study window occurred from February 2020 to April 2020, coinciding with the first stages of the COVID-19 pandemic, when change was rapidly occurring in many aspects of healthcare. Survey questions were devised and written prior to the onset of the pandemic, so specific impacts of COVID-19 were not assessed. We used chi-squared tests to test the associations between responses and burnout, after determining that clustering of responses within programs was not associated with significant variance when tested with generalized additive models. Data analysis was conducted using R 3.6 (R Core Team, Vienna, Austria).

## Results

Eleven programs enrolled in the study (26% of eligible programs), and responses were received from 30 of 34 eligible fellows (88% response rate) and 86 of 102 eligible faculty (84% response rate) at these 11 programs. The demographics of the faculty and fellow groups along with program characteristics are shown in Table 1. Fellows and faculty differed in mean age (faculty: 48 years, fellows: 32 years) and proportion married (84% faculty, 72% fellows) or with children (71% faculty, 56% fellows) but not in the percentage of US medical graduates (76% faculty, 72% fellows) or sex (female 58% faculty and 56% fellows). For fellows, there was a higher proportion of individuals with >$200,000 of educational debt and a lower proportion of those with no debt compared with faculty. In terms of academic rank, the majority of faculty were at the Assistant or Associate Professor levels and fellows were relatively evenly distributed across PGY year 4-6, with a small number of PGY7 fellows. Just under 20% of faculty worked less than 1.0 full time equivalent (FTE), compared to 7% of fellows.

**Table 1:** Fellow and faculty demographic characteristics.

	**Faculty (*n* = 86)**	**Fellows (*n* = 30)**
Age in years (mean, range)	47.7 (34-73)	32.2 (29-43)
Gender		
Female (*n*, %)	44 (51.2)	23 (76.7)
Male (*n*, %)	41 (47.7)	7 (23.3)
Prefer not to answer	1 (1.2)	0 (0)
Race/ethnicity		
Asian (*n*, %)	17 (20.7)	7 (23.3)
Black or African American (*n*, %)	1 (1.4)	1 (3.3)
Hispanic, Latino or of Spanish origin (*n*, %)	3 (3.7)	1 (3.3)
Multiple (*n*, %)	1 (1.2)	2 (6.7)
White (*n*, %)	57 (69.5)	17 (56.7)
NA or other (*n*, %)	3 (3.7)	2 (6.7)
IMG (*n*, %)	22 (26.2)	4 (13.3)
LGBTQ (*n*, %)	3 (3.5)	1 (3.3)
Educational debt, USD (*n*, %)		
None	50 (58.1)	13 (43.3)
< $50,000	8 (9.3)	0 (0)
$50,000–$99,000	5 (5.8)	1 (3.3)
$100,000–$199,000	16 (18.6)	2 (6.7)
$200,000–$299,000	5 (5.8)	9 (30)
>$300,000	2 (2.3)	5 (16.7)
Marital status		
Single	5 (5.8)	10 (33.3)
Married/partner	77 (89.5)	20 (66.7)
Divorced	2 (2.3)	0 (0)
Children		
Yes	66 (80.5)	11 (36.7)
No	16 (19.5)	19 (63.3)
Fellow level		
PGY4	n/a	7 (23.3)
PGY5	n/a	11 (36.7)
PGY6	n/a	9 (30)
PGY7 or higher	n/a	3 (10)
Faculty rank		
Instructor/acting asst prof	5 (6.1)	n/a
Assistant professor	34 (41.5)	n/a
Associate professor	25 (30.5)	n/a
Professor	18 (22)	n/a
FTE status		
1.0 FTE	69 (81.2)	25 (92.6)
<1.0 FTE	16 (18.9)	2 (7.4)
Burned Out (*n*, %)	14 (16.3)	4 (13.3)

The prevalence of burnout among both fellows and faculty was low (16.3% for faculty and 13.3% for fellows). There were no significant associations between burnout and the demographic characteristics listed in Table 1 (not shown). Table 2 evaluates the relationship between burnout and program-level characteristics, which were all non-significant.

**Table 2:** Program and work characteristics and burnout.

	**Without burnout (*n* = 98)**	**With burnout (*n* = 18)**	***P*-value**
Fellowship program size, *n* (%)			0.26
Small (1-3 fellows)	40 (40.8)	9 (50)	
Medium (4-6 fellows)	41 (41.8)	4 (22.2)	
Large (>7 fellows)	17 (17.3)	5 (27.8)	
Faculty division size, *n* (%)			0.63
Small (1-3 faculty)	6 (6.2)	2 (11.1)	
Medium (4-6 faculty)	7 (7.3)	2 (11.1)	
Large (>7 faculty)	83 (86.5)	14 (77.8)	
Current rotation/block^*^, *n* (%)			0.60
Inpatient	15 (18.3)	3 (18.8)	
Outpatient	25 (30.5)	4 (25)	
Both inpatient and outpatient	10 (12.2)	4 (25)	
Research	32 (39)	5 (31.2)	
Prior rotation/block^*^, *n* (%)			0.65
Inpatient	18 (20.2)	4 (25)	
Outpatient	25 (28.1)	4 (25)	
Both inpatient and outpatient	13 (14.6)	4 (25)	
Research	33 (37.1)	4 (25)	
FTE status, *n* (%)			0.94
<1.0	15 (16)	3 (16.7)	
1.0	79 (84)	15 (83.3)	
Co-fellow (same year of training)^†^, *n* (%)			0.97
Yes	17 (65.4)	2 (66.7)	
No	9 (34.6)	1 (33.3)	
Vacation (most recent), *n* (%)			0.41
Within past month	12 (12.2)	1 (5.6)	
>1 month ago	86 (87.8)	17 (94.4)	
Weekend off, *n* (%)			0.20
Previous weekend	67 (68.4)	15 (83.3)	
>1 week ago	31 (31.6)	3 (16.7)	

* *Faculty were asked to report what activity composed the bulk of their current or prior work weeks*.

† *Question completed by fellows only; asks if respondent has a co-fellow at the same level of training*.

Table 3 shows the relationship between personal characteristics and burnout using validated survey instruments. Summary measures from all these personal characteristics tools were significantly associated with burnout. Those individuals meeting criteria for burnout were more likely to report lower resilience, lower self-compassion, lower perceived quality of life, higher stress and more sleepiness.

**Table 3:** Association of burnout with personal characteristics.

	**Without burnout (*n* = 98)**	**With burnout (*n* = 18)**	***P*-value**
Self-compassion scale, mean (SD)	3.379 (0.567)	2.759 (0.480)	<0.001
Brief resilience scale, mean (SD)	3.677 (0.668)	2.917 (0.528)	<0.001
Perceived stress scale, mean (SD)	1.385 (0.727)	2.444 (0.560)	<0.001
Epworth sleepiness scale, mean (SD)	0.786 (0.507)	1.194 (0.508)	0.002
Quality of life rating, mean (SD)	7.943 (1.188)	5.533 (1.642)	<0.001

*Self-Compassion Scale range 1-5, higher scores better; Brief Resilience Scale range 1-5, higher scores better; Perceived Stress Scale range 0-4 (scaled from range 0-40), lower scores better; Epworth Sleepiness Scale range 0-3 (scaled from range 0-24), lower scores better; Quality of Life Rating range 1-10, higher scores better*.

Most individual perceptions of workplace characteristics were associated with burnout (Table 4). Perceived lack of supportive and collaborative environments were associated with burnout. Less satisfaction with support from family, friends, faculty and colleagues were each separately associated with higher burnout levels. There was no significant difference between the two groups in the proportion reporting a high level of satisfaction from a spouse/partner. Fellows and faculty who perceived having adequate staffing and autonomy at work were less likely to be burned out.

**Table 4:** Faculty and fellow attitudes, job satisfaction, and burnout.

	**Without burnout (*n* = 98)**	**With burnout (*n* = 18)**	***P*-value**
Perception of autonomy at work *N* (%) definitely agree	27 (27.8)	1 (5.6)	0.043
Feel confident in financial knowledge and security			
*N* (%) definitely agree	49 (50)	3 (16.7)	0.009
Feel stress about money and finances *N* (%) definitely disagree	22 (22.7)	3 (16.7)	0.570
Endorse concern for burnout, *N* (%) yes			
Among fellows	20 (20.6)	7 (38.9)	0.093
Among faculty	32 (33)	16 (88.9)	<0.001
Among staff	35 (36.1)	9 (50)	0.265
Adequate staffing (physician and non-physician) *N* (%) strongly agree	38 (38.8)	1 (5.6)	0.006
Institution prioritizes and promotes physician wellness *N* (%) strongly agree	32 (33)	1 (5.6)	0.018
Career choice (PN) satisfaction *N* (%) very satisfied	65 (66.3)	4 (22.2)	<0.001
Work life balance satisfaction *N* (%) very satisfied	27 (27.6)	0 (0)	<0.001
Very satisfied with support from, *N* (%):			
Family	65 (67)	6 (33.3)	0.007
Spouse/significant other	61 (66.3)	10 (58.8)	0.552
Friends	57 (58.8)	5 (27.8)	0.015
Faculty	41 (43.6)	0 (0)	<0.001
Colleagues	54 (56.2)	1 (5.6)	<0.001
Attitudes toward learning environment			
*N* (%) strongly agree			
Collaborative rather than competitive	54 (55.7)	1 (5.6)	<0.001
Fellow education is a high priority	49 (50.5)	6 (33.3)	0.180
Career mentoring is a high priority	38 (39.2)	5 (27.8)	0.359

In fellows and faculty who endorsed burnout there was a lower proportion of respondents who were very satisfied with both career choice (non-burned out–66% vs. burned out−22%) and work-life balance (non-burned out–28% vs. burned out–0%). When asked about general perceptions of burnout among different groups in the healthcare setting, a high proportion of those meeting criteria for burnout reported concern for burnout among faculty (89% in those with burn out vs. 33% among those not burned out) whereas concern for burnout in fellows and staff was not significantly different between those who were burned out vs. those who were not burned out. Of note, while high satisfaction with work-life balance was endorsed by only 28% without burnout, no faculty/fellows with burnout were highly satisfied with their work-life balance. We found no relationship between the proportion of faculty burnout at an institution and the proportion of fellows burned out at that same institution.

## Discussion

Based on pilot data from 11 US-based programs, the prevalence of burnout among Pediatric Nephrologists is markedly lower than that reported among US physicians in general and lower than prior reported rates among pediatric residents. While this could be unique to pediatric nephrology and indicate a relative strength within the specialty, the study window occurring at the onset of the COVID-19 pandemic must be taken into consideration. A similar study conducted among nephrology trainees in 2020 (both adult and pediatric fellows) also demonstrated an unexpected low level of burnout at 15% (9). The longitudinal Pediatric Residency Burnout-Resilience Consortium Studyalso found a decrease in burnout in pediatric residents in 2020 [burnout rate in pediatric residents 31% in 2020 vs. 40% in 2019 and 52% in 2018, presentation by Zuniga et al. (10) PAS].

Research on the emotional response of individuals faced with crisis situations shows a distinct pattern of stages throughout the crisis. The initial phases, termed “the heroic” and “the honeymoon” phases occur when individuals work together to address a crisis or disaster, and are followed by other phases characterized by more negative responses (11). Since this survey was underway during the first COVID-19 pandemic in the US, we should consider these respondents as potentially influenced by this emotional framework. Though they were exposed to tremendous stress and uncertainty, they may also have been influenced by an expanded sense of purpose and meaning in their professional work in the early stages of the crisis. We can only surmise that the timing of the survey may have impacted the reported burnout rate. Interestingly, the relationships of personal characteristics and the impact of a supportive work environment did match the associations with burnout described in studies conducted in pre-COVID times in pediatric trainees (3, 4, 7). It remains to be seen if similar rates of burnout will be detected in these groups in non-COVID times.

An important finding of our study was that, among all the factors considered, burnout most closely associated with personal characteristics and perceptions of the workplace environment. Our study did not find any association among either individual demographic characteristics (such as academic rank, FTE, age, or sex) or program-level characteristics (such as program size, number of faculty, current or recent rotation/work activity) with burnout. These findings were consistent among both fellows and faculty. These findings seem to highlight that physician burnout can affect anyone and mitigation strategies could potentially be applied broadly (11). Prevalence of faculty burnout was not significantly associated with fellow burnout, but given our small sample size and low overall prevalence of burnout, the study may have been underpowered to detect such a relationship.

The high proportion of respondents with burnout reporting concern regarding faculty burnout is further suggestive of the impact the culture of the learning and working environment can have on individuals, both trainees and faculty. These ideas have been explored in a number of recent cross-sectional studies by Dyrbye et al. looking at resident perceptions of faculty professional behavior and relationships with trainees, program leadership qualities, level of trainee autonomy, and the relationship to burnout (12, 13). At least one longitudinal study found that burnout in the third year of residency was higher among women residents compared to men, with women reporting higher levels of negative interpersonal experiences and unfair treatment in prior years (14).

Despite these findings, our study does have some limitations. Although the participation among faculty and fellows at enrolled programs was quite high (>80%), the number of eligible programs enrolled in the survey was low (26%). It is unclear if the individuals at the enrolled programs are a representative sample of the specialty as a whole, though the high participation rate within programs is a strength. Additionally, due to the cross-sectional nature of the study, causation between the reported factors and burnout cannot be inferred. Lastly, we did not have a sufficient sample size to explore meaningful associations between faculty burnout and fellow burnout.

Given the high prevalence of burnout among physicians in general, interventions to address and prevent burnout must take a multi-tiered approach. It is simply not adequate to place the burden of burnout solely on the individual. Systemic-level changes and interventions are equally important (4, 6, 10, 15). Our study findings suggest many opportunities to modify the workplace environment to help with burnout. Fostering a collaborative workplace environment with supportive peers and mentors is something that can be pursued at division, departmental, and institution levels. Other areas of intervention could include: creating opportunities for increased physician autonomy within large healthcare organizations and university systems, innovative approaches to ensure adequate staffing and changing the work culture to allow flexibility in scheduling to promote successful work/life integration. Future studies are needed to follow trends in burnout among pediatric nephrologists, particularly with the rapidly changing environment created by the COVID-19 pandemic. Broadening studies to include other pediatric sub-specialties will also be helpful to compare and identify differences, as prior studies show considerable variability among specialties, though pediatrics is typically all grouped together (2).

## Data Availability Statement

The datasets presented in this article are not readily available because data are stored with the Association of Pediatric Program Directors Research Organization and are only available to member institutions. Requests to access the datasets should be directed to susan.halbach@seattlechildrens.org.

## Ethics Statement

The studies involving human participants were reviewed and approved by Seattle Children's Hospital. Written informed consent for participation was not required for this study in accordance with the national legislation and the institutional requirements.

## Author Contributions

SH, PS-M, KP, JM, and AS: study concept and design and statistical analysis. SH: manuscript preparation. PS-M, KP, JM, DW, and AS: manuscript review and revision. All authors contributed to the article and approved the submitted version.

## Conflict of Interest

The authors declare that the research was conducted in the absence of any commercial or financial relationships that could be construed as a potential conflict of interest.

## Publisher's Note

All claims expressed in this article are solely those of the authors and do not necessarily represent those of their affiliated organizations, or those of the publisher, the editors and the reviewers. Any product that may be evaluated in this article, or claim that may be made by its manufacturer, is not guaranteed or endorsed by the publisher.
